# Critical role for uricase and xanthine dehydrogenase in soybean nitrogen fixation and nodule development

**DOI:** 10.1002/tpg2.20172

**Published:** 2021-12-13

**Authors:** Cuong X. Nguyen, Alice Dohnalkova, C. Nathan Hancock, Kendall R. Kirk, Gary Stacey, Minviluz G. Stacey

**Affiliations:** ^1^ Division of Plant Sciences Univ. of Missouri Columbia MO 65211 USA; ^2^ Environmental Molecular Sciences Laboratory Pacific Northwest National Laboratory Richland WA 99354 USA; ^3^ Dep. of Biology & Geology Univ. of South Carolina Aiken SC 29801 USA; ^4^ Edisto Research & Education Center Clemson Univ. Blackville SC 29817 USA; ^5^ Division of Biochemistry Univ. of Missouri Columbia MO 65211 USA

## Abstract

De novo purine biosynthesis is required for the incorporation of fixed nitrogen in ureide exporting nodules, as formed on soybean [*Glycine max* (L.) Merr.] roots. However, in many cases, the enzymes involved in this pathway have been deduced strictly from genome annotations with little direct genetic evidence, such as mutant studies, to confirm their biochemical function or importance to nodule development. While efforts to develop large mutant collections of soybean are underway, research on this plant is still hampered by the inability to obtain mutations in any specific gene of interest. Using a forward genetic approach, as well as CRISPR/Cas9 gene editing via *Agrobacterium rhizogenes*‐mediated hairy root transformation, we identified and characterized the role of GmUOX (Uricase) and GmXDH (Xanthine Dehydrogenase) in nitrogen fixation and nodule development in soybean. The *gmuox* knockout soybean mutants displayed nitrogen deficiency chlorosis and early nodule senescence, as exemplified by the reduced nitrogenase (acetylene reduction) activity in nodules, the internal greenish‐white internal appearance of nodules, and diminished leghemoglobin production. In addition, *gmuox1* nodules showed collapsed infected cells with degraded cytoplasm, aggregated bacteroids with no discernable symbiosome membranes, and increased formation of poly‐β‐hydroxybutyrate granules. Similarly, knockout *gmxdh* mutant nodules, generated with the CRISPR/Cas9 system, also exhibited early nodule senescence. These genetic studies confirm the critical role of the de novo purine metabolisms pathway not only in the incorporation of fixed nitrogen but also in the successful development of a functional, nitrogen‐fixing nodule. Furthermore, these studies demonstrate the great utility of the CRISPR/Cas9 system for studying root‐associated gene traits when coupled with hairy root transformation.

## Core Ideas

- GmUOX1 and GmXDH1 are essential for successful nodule development and N fixation in soybean roots.
- Purine catabolism impacts the plant defense response related to nodule maintenance in soybean.
- CRISPR/Cas9 system with hairy root transformation offers method for studying root‐associated gene traits.

**Abbreviations**
CGH: comparative genomic hybridization
dpi: days after inoculation
FN: fast neutron
GFP: green fluorescent protein
gRNA: guide RNA
PCR: polymerase chain reaction
PHB: poly‐β‐hydroxybutyrate
qRT‐PCR: quantitative real‐time–polymerase chain reaction
sgRNA: single‐guide RNA
TEM: transmission electron microscopy
UOX: uricase
XDH: xanthine dehydrogenase

## 1 INTRODUCTION

Soybean [*Glycine max* (L.) Merr.] is one of the most valuable crops for seed protein and oil production and is capable of symbiotic nitrogen fixation. A variety of genetic and genomic resources are available for soybean including the completed genome sequence, gene expression atlas, as well as extensive proteomic and metabolomics studies, which support the notion that soybean is a feasible legume model plant (Brechenmacher et al., 2012; Libault et al., 2010; Schmutz et al., 2010; Valdés‐López et al., 2016). However, the highly duplicated genome—where ∼75% of predicted genes are present in multiple copies—presents a challenge for studies on gene function (Schmutz et al., 2010). Mutagenesis has played an important role in breeding and functional genomics over the past three decades. Several soybean mutant populations have been developed, using different physical, chemical, and transposon mutagenesis, to investigate soybean gene function especially for those encoding important agronomic traits (Campbell & Stupar, 2016).

Fast neutron (FN) irradiation was shown to be an effective mutagen to induce genomic deletions and duplications in plants (Hoffmann et al., 2007; Li et al., 2001; Oldroyd & Long, 2003). Soybean FN mutant populations have been developed in the line M92‐220 and the cultivar Williams 82 to serve as genetic resources where genetic lesions can be easily detected using whole‐genome microarray hybridization or comparative genomic hybridization (CGH) (Bolon et al., 2011, 2014; Stacey et al., 2016). For example, these two soybean FN populations have proven to be useful genetic resources for identifying genes involved in seed weight, seed composition, trichome development, and altered nodule morphology (Campbell et al., 2016; Gillman et al., 2014; Nguyen et al., 2021; Stacey et al., 2016; Vincent et al., 2015). Besides their use for forward genetic screens, FN mutagenized populations have been successfully developed as reverse genetic platforms for *Arabidopsis thaliana* (L.) Heynh. and barrel clover (*Medicago truncatula* Gaertn.) using polymerase chain reaction (PCR)‐based screening (Li et al., 2001; Rogers et al., 2009). However, soybean FN populations have not been extensively exploited for reverse genetic screening. More than 350 soybean FN mutant lines have been genotyped by CGH, in which ∼15,000 genes were detected within homozygous and hemizygous deletion regions (Bolon et al., 2014). This valuable CGH genotype data will expand the ability of soybean researchers to obtain useful mutants in their specific gene of interest.

**Core Ideas**

- GmUOX1 and GmXDH1 are essential for successful nodule development and N fixation in soybean roots.
- Purine catabolism impacts the plant defense response related to nodule maintenance in soybean.
- CRISPR/Cas9 system with hairy root transformation offers method for studying root‐associated gene traits.

Nodulation is the result of a symbiotic interaction between legume plants and soil bacteria (rhizobia), which provides the plant with a source of nitrogen and contributes to the global use of nitrogen in ecological and agricultural systems (Herridge et al., 2008; Vance, 2001). Nodule formation is a complex and multistep process that requires the mutual exchange of molecular signals between the symbiont and plant host (Roy et al., 2020; Udvardi & Poole, 2013). In soybean, atmospheric di‐nitrogen (N_2_) is fixed to form ammonia by bacterial nitrogenase in the infected nodule cell. Ammonia is then released into the cytosol of the infected cell, reduced to purine nucleotides, and incorporated into ureides (allantoin and allantoate), the major transport form of nitrogen in nitrogen‐fixing soybean (Smith & Atkins, 2002; Tegeder, 2014; Udvardi & Poole, 2013; Werner & Witte, 2011). The ureide biosynthetic pathway begins with the degradation of purine bases to xanthine, which is then oxidized to uric acid via xanthine dehydrogenase (XDH). These processes take place in the cytosol of the infected cell of the nodule (Smith & Atkins, 2002). Uric acid is transferred to uninfected cells and imported into the peroxisomes where uricase (UOX) oxidases uric acid to form ureides (allantoin and allantoic acid). Following synthesis, the ureides are transported to the nodule vasculature and exported to other parts of the plant through the xylem (Supplemental Figure S1) (Collier & Tegeder, 2012; Smith & Atkins, 2002; Tegeder, 2014; Werner & Witte, 2011). Although the presence of the ureide biosynthesis pathway is well documented in legume plants, identification of the enzymes involved is largely based on predicted genome annotations with little direct genetic or biochemical evidence to confirm such predictions.

In the current work, we used soybean FN mutagenesis and CRISPR/Cas9 to generate *gmuox* (*uricase*) and *gmxdh* (*xanthine dehydrogenase*) mutants of soybean. Mutation of *gmuox* led to the accumulation of uric acid, as well as the production of early senescent nodules that are unable to fix nitrogen (Fix^−^). A similar Fix^−^ phenotype was also observed in the *gmxdh* mutant soybean nodules. These genetic studies confirm the critical role of the ureide biosynthetic pathway in symbiotic nitrogen assimilation but also point to a critical role for the pathway in supporting the normal development of a functional, nitrogen‐fixing nodule. The data presented also demonstrate the great utility of the CRISPR/Cas9 system for studying root‐associated gene traits when coupled with hairy root transformation in soybean.

## 2 MATERIALS AND METHODS

### 2.1 Plant materials, growth conditions, and root nodule formation

Soybean seeds of cultivar Williams 82 and its mutant lines FN 6‐12 (FN300081.02.01.M4) and K14 (FN201610.01.01.M4) (https://soybase.org/mutants/) were used for nodulation screening. Soybean seeds were surface sterilized with 70% ethanol for 1 min, followed by 10% Clorox for 10 min. They were then rinsed six times with sterile deionized water and germinated in 7.62‐cm pots (3” pots; three seeds per pot) filled with 1:1 (v/v) mixture of sterilized perlite and vermiculite (Hummert International). Pots were watered regularly with nitrogen free plant nutrient solution B&D (Broughton & Dilworth, 1971) and maintained in an environmentally controlled plant growth chamber (16:8 h, light/dark photoperiod; 27 °C; 80% humidity). Microsymbiotic nitrogen‐fixing, *Bradyrhizobium japonicum* USDA110 was cultured in HM medium (Cole & Elkan, 1973) with 0.004% chloramphenicol and grown at 30 °C for 3 d. After 3 d of culture, bacteria were pelleted and diluted in sterile water to an optical density (OD_600_) of 0.2 for inoculation. Bacterial solution was inoculated directly onto the soybean seed at the time of sowing. For growth and nodulation under nitrogen supplementation, 5 mM urea was added to BD solution and plants were grown and nodulated as described above.

Genetic backcrosses were performed by pollinating emasculated flowers of the parental cultivar Williams 82 with pollen from mutant line FN 6‐12 grown at the Bradford Research and Experiment Center, University of Missouri, Columbia. Growth and phenotypic observations of BC_1_F_2_ plants were done in the green house, Bond Life Science Center, University of Missouri, Columbia, using the same methods described above. Crown nodules at 30 d after inoculation (dpi) were collected and scored for segregation of nodulation phenotype.

### 2.2 Vector construction and soybean hairy root transformation and nodulation

The human codon‐optimized *Cas9* gene, driven by the *CaMV 35S* promoter, and the chimeric single guide RNA, driven by the *AtU6‐26* promoter, were gifts from Jian‐Kang Zhu (Feng et al., 2013). The binary vector pCAMGFP‐CsVMV::GWOX was used as vector backbone for soybean hairy root transformation because the T‐DNA of this vector contained a constitutively expressed green fluorescent protein (GFP) cassette, which enabled identification of transformed roots by GFP fluorescence (Li et al., 2010).

Soybean‐specific, single‐guide RNA sequences were designed using the web tool CCTop (Stemmer et al., 2015). Two single‐guide RNAs (sgRNAs) (sgRNA1 and sgRNA2) were used to create defined deletions within each target gene coding sequence. The selected dual sgRNA target sequences were located in the exons of *GmUOX1* and *GmXDH1* and the target protospacer adjacent motif sites were separated by ∼240 and 236 bp for *GmUOX1* and *GmXDH1*, respectively. The designed oligonucleotides (20‐bp targeting sequences) were synthesized by Integrated DNA Technologies, Inc. (Coralville, IA) then annealed to generate dimers and cloned into *BbsI* sites of pSK‐AtU6‐26 to create pSK‐AtU6‐26‐sgRNA. These plasmids were confirmed by Sanger sequencing. Oligos for each target of *GmUOX1* and *GmXDH1* are listed in Supplemental Table S1. To obtain functional Cas9 expression constructs for targeted mutagenesis, pSK‐AtU6‐26‐sgRNA1 was cut with *BamHI‐SpeI*, pSK‐AtU6‐26‐sgRNA2 was cut with *BamHI‐PstI*, and pSK‐35S‐Cas9 was cut with *KpnI‐SpeI*. These three fragments were assembled into pCAMGFP‐CsVMV:GWOX by *KpnI‐PstI* ligation. The positive plasmids were confirmed by Sanger sequencing then were introduced into *Agrobacterium rhizogenes* K599 by electroporation and used for hairy root transformation as described by Kereszt *et al*. (2007). Briefly, 3.5‐d‐old soybean seedlings (Williams 82) were infected by *A. rhizogenes* K599 harboring the *Cas9* expression constructs for the target genes. Twelve days after infection, roots were observed under a Leica M205FA stereomicroscope and nontransgenic hairy roots, that is, those not expressing GFP, were removed using a razor blade. Transgenic composite plants were then inoculated with *B. japonicum* USDA110. Nodules produced on transgenic roots were harvested at 28 dpi, confirmed for strong GFP expression, and observed for nodulation phenotype.

### 2.3 RNA isolation, complementary DNA synthesis, and transcript‐level analysis

RNA extraction was performed using TRIzol reagent (15596026, Invitrogen) following the manufacturer's instructions. Complementary DNA was synthesized using oligo(dT) primers (15‐mer) and M‐MLV reverse transcriptase enzyme (M1701, Promega) following the manufacturer's instructions. Gene transcript levels were determined by quantitative real‐time–polymerase chain reaction (qRT‐PCR) using an ABI17500 real‐time PCR following the SYBR Green method (4309155, Applied Biosystems). Gene expression levels were normalized to the expression of the soybean housekeeping genes *cons6* and *cons4* (Libault et al., 2008). Data and statistical analysis were performed using QbasePLUS software (Biogazelle) (Hellemans et al., 2007). Primers used for qRT‐PCR were listed in Supplemental TableS1.

### 2.4 DNA extraction, PCR assay, and sequencing

Chromosomal DNA was isolated following routine isolation techniques from young leaf tissues or hairy roots for genotyping of FN mutants (FN 6‐12 and K14 F) or CRISPR/Cas9 transgenic hairy roots, respectively. The PCR amplification was performed using gene‐specific primers with Q5 high fidelity DNA polymerase (M0491L, NEB). Primers used for genetic mapping and CRISPR genotyping were listed in Supplemental TableS1. The PCR products were extracted from the gel, purified using Wizard SV Gel and PCR Clean‐Up System (PRA9282, Promega), and cloned into the pGEMT‐easy vector (PRA1360, Promega). Five individual clones were sequenced by Sanger sequencing using the M13‐F and M13‐R primers. Sequence alignments were done using MUSCLE web tool (http://www.ebi.ac.uk/Tools/msa/muscle/).

### 2.5 Histology and microscopy

Excised nodules were either hand sectioned or sectioned to 80 μm thickness using a VT 1000S vibratome after embedding with 2% agarose. These sections were used to visually observe or image nodule color using a Leica M205FA stereomicroscope.

To analyze the nodule structure, the nodules were fixed in formaldehyde–acetic acid–alcohol (10:5:50) solution. The fixed samples were sent to IDEXX Laboratories, Inc. for paraffin embedding and sectioning to 10 μm thickness with a microtome. After sectioning, microtome sections were dewaxed using Citrisolv (04‐355‐121 FisherBrand), dehydrated, stained with 1% toluidine blue, and examined with an Olympus Vanox AH‐3 microscope.

For transmission electron microscopy (TEM), nodules were processed and imaged as previously described (Gillman et al., 2014). In brief, slices of nodule tissue were high‐pressure frozen (BAL‐TEC HPF 010), freeze substituted for 5 d at −90 °C in acetone containing 2% osmium tetroxide, warmed and embedded in Spurrs resin, and thin sections were stained with uranyl and lead salts followed by imaging using a LEO912 AB energy filter TEM. All microscopic examinations were done on excised nodules 4 wk after inoculation. These morphological evaluations were done on nodules formed on older roots, that is, on tap roots ∼2 cm from the stem–root junction and on lateral roots formed within this region. This was done to make sure that nodules were of a similar developmental stage when evaluated.

### 2.6 Quantification of uric acid and xanthine

Xanthine and uric acid were extracted by grinding frozen tissues following the method as described by Hauck et al. (2014). Briefly, 50 mg of freshly ground nodule tissues was added to 500 μl of 0.1 M Tris, pH 7.5, incubated at 95 °C for 10 min, then centrifuged at room temperature at 20,000 × *g* for 15 min. Pellets were re‐extracted one more time and supernatants were pooled. The pooled supernatant was centrifugated at 18,000 × *g* for 15 min at room temperature to remove tissue debris and the supernatant was used to measure the uric acid or xanthine content. Uric acid and xanthine were quantified using Amplex Red Uric Acid/Uricase Assay Kit (A22181, Invitrogen) and Amplex Red Xanthine/Xanthine Oxidase Assay Kit (A22182, Invitrogen) following the manufacturer's instructions. Absorbance at 560 nm was determined using a Synergy Multi‐Mode Microplate Reader (BioTek Instruments).

### 2.7 Acetylene reduction assay

Four‐week‐old soybean crown nodules were harvested for acetylene reduction assay. Briefly, ∼400 mg detached nodules from wild‐type and FN 6‐12 were collected, placed in sealed 60‐ml vials, and incubated with 10% acetylene at room temperature for 1 h. After incubation, 1 ml of gas sample was removed from each sealed vials using 1‐ml syringe and injected into a gas chromatograph (TRACE 1300, TG‐BOND Q^+^ column, Thermo Scientific). Known amounts of ethylene were also ran in the gas chromatograph as standards for calculating the amounts of ethylene produced in the sample vials. For each sample, the area of the obtained peak of ethylene was recorded, and the acetylene reduction was calculated as the amount of ethylene produced per hour and mass of nodules. Four biological replications were performed for each genotype.

### 2.8 Leaf nitrogen and leghemoglobin quantification

Leaf nitrogen content was measured in inoculated plants grown in a sandy, unfertilized field at the Clemson University Edisto REC in Blackville, SC. Plots were 3 m long with 96‐cm row spacing and arranged in four complete randomized replicates. The most recent fully expanded trifoliates were collected at R1 stage (47 d after planting) and analyzed for nitrogen content using the Kjehldahl method at the Clemson University Agricultural Service Laboratory.

Leghemoglobin content was determined using published protocol (LaRue & Child, 1979). Briefly, 100 mg of fresh nodule tissues was homogenized in 2 ml solution of 0.02% (w/v) potassium‐ferricyanide and 0.1% sodium bicarbonate in a chilled mortar. Water‐saturated oxalic acid was purified several times by boiling in a large beaker in a fume hood and then redissolved in distilled water. A 100‐μl nodule extract was added to 2 ml of water‐saturated oxalic acid in screw‐capped tubes and autoclaved for 30 min at 120 °C. Fluorescence was measured with a Synergy Multi‐Mode Microplate Reader (BioTek Instruments) set at 405 nm for excitation and 650 nm for emission wavelengths. Bovine hemoglobin (H2500, Sigma‐Aldrich) was used as a standard.

### 2.9 Statistical analysis

Sample means between genotypes or treatments were compared using Student's *t* test or one‐way ANOVA followed by Tukey's HSD test. All statistical analyses were performed using SPSS (IBM SPSS v23).

## 3 RESULTS

### 3.1 Identification of soybean *URICASE1* (*GmUOX1)* FN mutants

**FIGURE 1:**
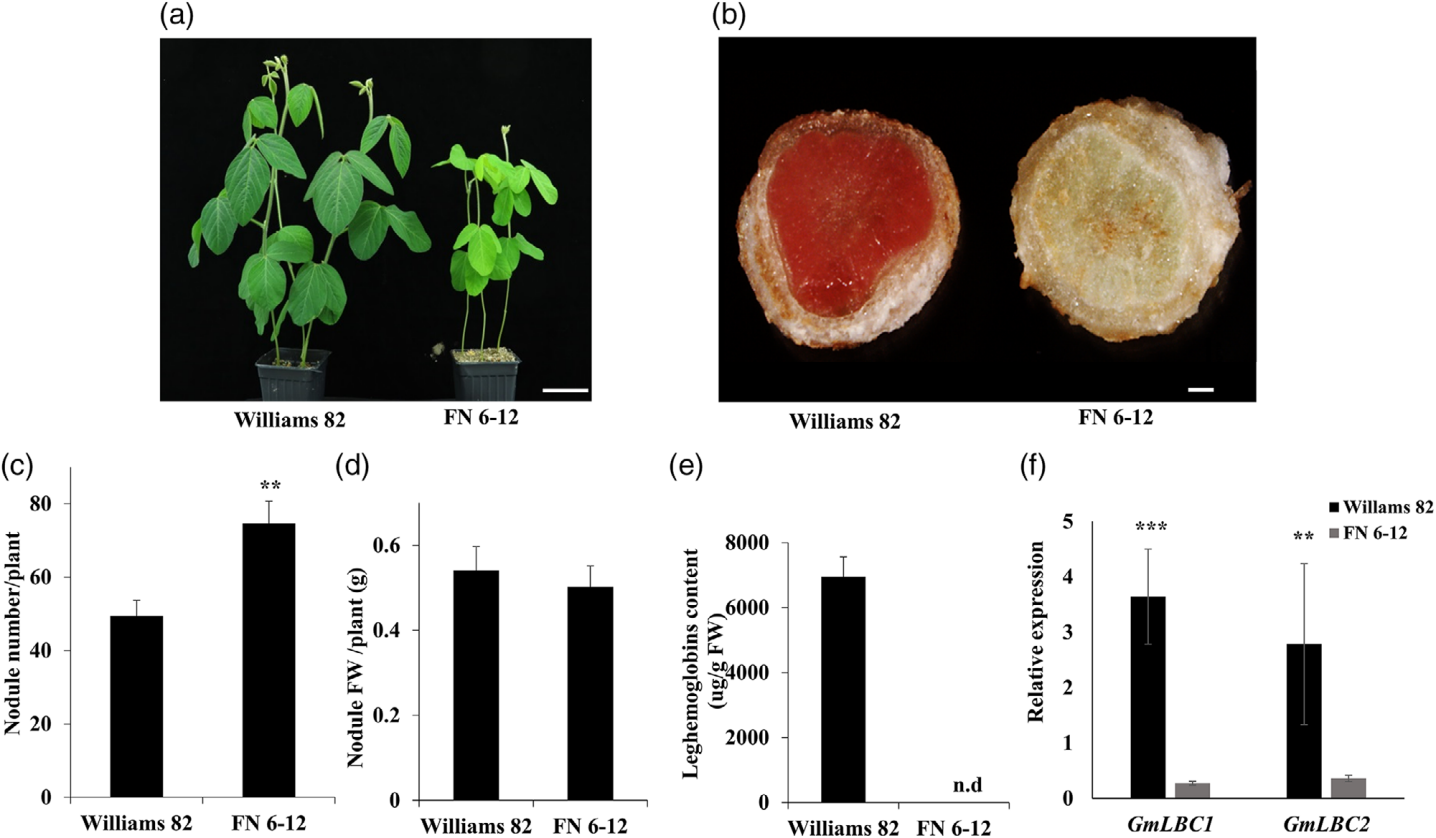
Symbiotic phenotypes of the mutant line FN 6‐12 at 30 d after inoculation with *B*. *japonicum* strain USDA 110. (a) Representative photograph of ‘Williams 82’ (wild type) and FN 6‐12 at V3 stage of vegetative growth showing stunted growth and leaf chlorosis of the mutant under symbiosis nutrition condition. (b) Representative photograph of Williams 82 (wild type) and FN 6‐12 showing pink and white nodule coloration, respectively. (c) Nodule numbers and (d) nodule fresh weight in the mutant line and wild type Williams 82 at 30 d after inoculation (dpi) (*n* = 18). Data represent means ± SD. (e) Leghemoglobin content and (f) expression levels of *GmLBC1* and *GmLBC2* in Williams 82 and FN 6‐12 nodules at 30 dpi (*n* = 4). Data represent means ± SD. ** and *** indicate significant differences (by Student's *t* test) between the two genotypes *P* < .01 and *P* < .001, respectively. n.d., not detectable. Bars, (a) 5 cm; (b) 300 μm

To facilitate gene discovery in soybean, we developed a large collection of mutants using FN irradiation in cultivar Williams 82. We screened ∼2,000 field‐grown FN lines for visual phenotypes such as leaf chlorosis. Further screening of chlorotic mutants identified two independent mutant lines, FN 6‐12 and K14, that showed reduced growth and chlorotic leaves under conditions of symbiotic nitrogen fixation at 30 dpi (Figure 1a; Supplemental Figures S2a and S2b). Mutant plants also produced white nodules rather than the pink or light pink color associated with effective, nitrogen‐fixing nodules (Fix^+^) on wild‐type roots (Figure 1b; Supplemental Figures S2c andS2d). White nodules were mostly located near the root crown, while pink nodules were found mostly on younger lateral and tap roots of the FN mutant lines. We observed a 1.5‐fold increase in nodule number in mutant roots compared with wild‐type roots (Figure 1c). However, no difference in nodule fresh weight was observed between the two genotypes (Figure 1d).

The pink coloration in Fix^+^ nodules is due to the production of high amounts of leghemoglobin, an O_2_–binding hemeprotein. Leghemoglobin maintains the low O_2_ cellular environment required for nitrogenase activity, while facilitating the flux of O_2_ toward respiration sites in infected nodule cells (Appleby, 1984; Downie, 2005). The production of leghemoglobin is essential for effective N_2_ fixation, and its diminished accumulation, in white nodules, is associated with a Fix^−^ phenotype (Ott et al., 2009; Ott et al., 2005). To confirm that white nodules indeed contain low amounts of leghemoglobin (i.e., are Fix^−^), we compared leghemoglobin levels in wild‐type and mutant white nodules formed at 30 dpi. We found high levels of leghemoglobin in wild‐type nodules, whereas very low or undetectable levels were observed in white nodules at 30 dpi (Figure 1e). Likewise, the expression levels of the leghemoglobin genes *GmLBC1* (*Glyma10g34280*) and *GmLBC2* (*Glyma10g34290*) were significantly decreased in the FN nodules compared with wild‐type nodules (Figure 1f)

**FIGURE 2:**
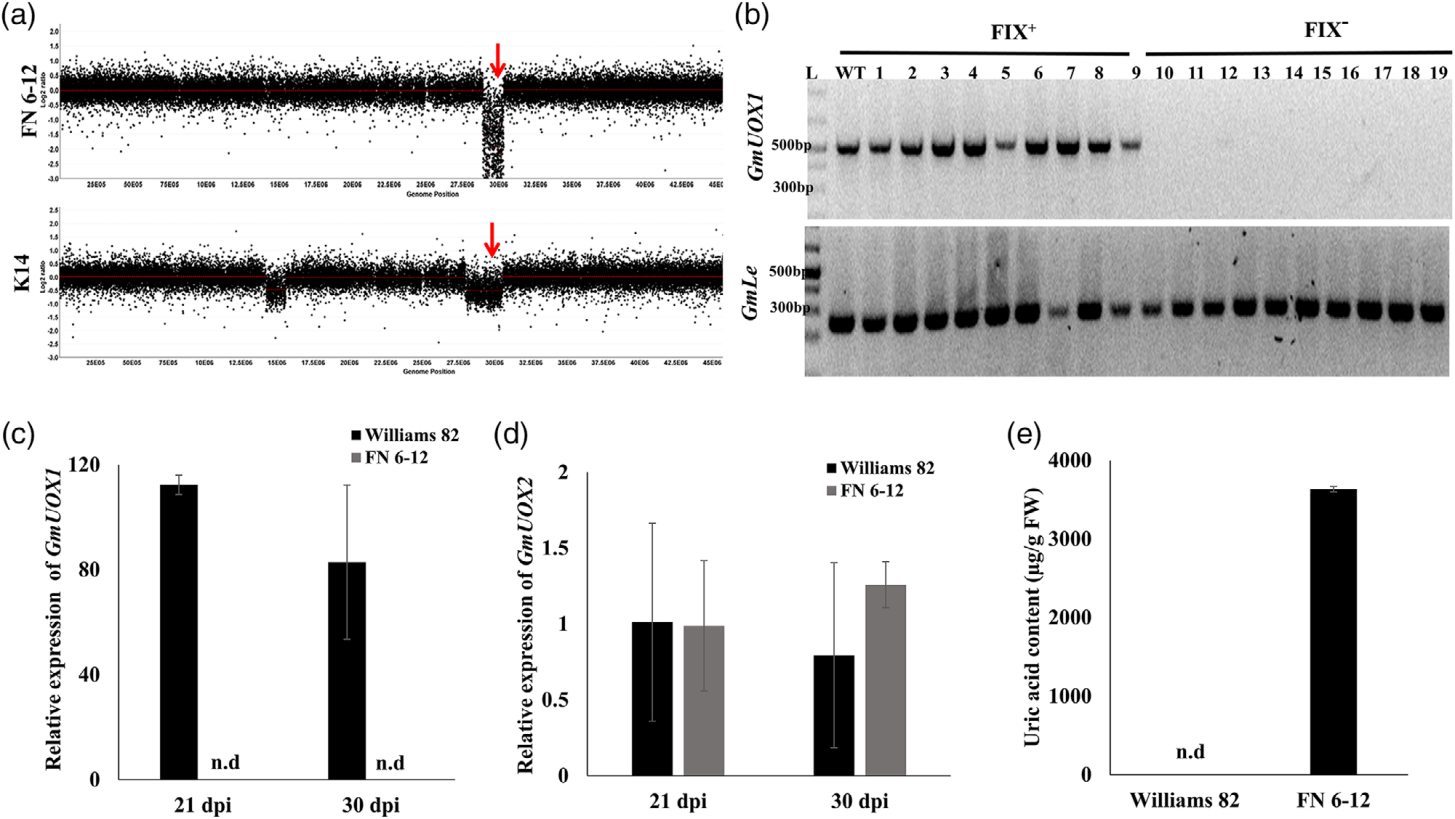
Identification of induced genetic deletions in mutant line FN 6‐12 and K14 and cosegregation of Fix^−^ nodule phenotypes with chromosome 10 deletion. (a) Comparative genome hybridization output for chromosome 10 of mutant lines FN 6‐12 (upper panel) and K14 (lower panel). The overlapping deletions encoding the soybean *Uricase1* gene (*Glyma10g23790*) is indicated by the red arrows. (b) Polymerase chain reaction (PCR) amplification assay for detecting soybean *Uricase 1* (*GmUOX1*, upper panel) gene deletion in BC_1_F_2_ FN 6‐12 plants. Lanes: L, molecular weight ladder; 1, chromosomal DNA from ‘Williams 82’ (wild‐type); 2‐9, chromosomal DNA from Fix^+^ BC_1_F_2_ FN 6‐12 plants; 10‐19, chromosomal DNA from Fix^−^ BC_1_F_2_ FN 6‐12 plants. *Glyma.02g012600* (*GmLe*, lower panel) encodes a soybean lectin domain protein was used as positive control PCR for the chromosomal DNA. (c) Expression levels of *GmUOX1*, (d) *GmUOX2*, and (e) uric acid levels in nodules of wild type Williams 82 and the mutant line FN 6‐12 at 30 d after inoculation. Data represent means ± SE [(c, d) *n* = 4; (e), *n* = 6] *** indicates significant differences (*t* test) between two genotypes at *P* < .001. FW, fresh weight; n.d., not detectable

Based on our CGH analysis (CGH data are available at https://soybase.org/mutants), the mutant lines FN 6‐12 (FN300081) and K14 (FN201610) harbor eight and six deletions, respectively. Interestingly, both mutants harbor overlapping deletions in chromosome 10, indicating that the causative gene for the observed nodule phenotype is likely encoded within this overlapping deleted region (Figure 2a). Based on the soybean *Gmax v.1.1* annotation (https://soybase.org), the overlapping deleted region in chromosome 10 encodes 36 genes, only one of which, *Glyma10g23790*, is exclusively expressed in the nodule (Supplemental Figure S3). *Glyma10g23790* (*GmUOX1*) is one of the two predicted *URATE OXIDASE* or *URICASE* genes involved in the production of ureides, the form of fixed nitrogen produced in soybean nodules (Suzuki & Verma, 1991; Werner & Witte, 2011). A second *GmUOX* gene, *Glyma20g17440* (*GmUOX2*) whose encoded protein shares 99% identical protein sequence to GmUOX1, is expressed at low levels in various soybean tissues, including nodules (Supplemental Figure S3). We backcrossed mutant FN 6‐12 to wild‐type Williams 82 and examined the segregation of the Fix^−^ phenotype in four independent BC_1_F_2_ families. We found that BC_1_F_2_ progenies segregated at a 3:1 ratio of wild type to Fix^−^ phenotypes, indicating that the nodule phenotype is due to a single recessive locus (χ^2^ = 0.041, *P* = .839; Supplemental Table S2). To determine if the *GmUOX1* deletion cosegregates with the Fix^−^ phenotype, BC_1_F_2_ plants derived from mutant FN 6‐12 were genotyped by PCR amplification of *GmUOX1*. We found that *GmUOX1* deletion indeed cosegregated with the Fix^−^ phenotype (Figure 2b). K14 plants homozygous for the defective nodulation phenotype produced very few seeds, most likely because of second‐site mutations causing abnormal flower production (Supplemental Figure S2e). Therefore, line K14 was not backcrossed, and further phenotypic characterization of *gmuox1* nodule development was done on line FN 6‐12.

To determine if *GmUOX1* is indeed deleted in the mutant FN 6‐12, as indicated by our CGH analysis as well as to ascertain that *GmUOX1* encodes the major UOX isoform in soybean nodules, we determined the relative expression of *GmUOX1* and *GmUOX2* in wild‐type and mutant nodules by qRT‐PCR. We found that *GmUOX1* showed 111‐fold higher expression than Gm*UOX2* in wild‐type nodules, indicating that *GmUOX1* encodes the major isoform expressed in soybean nodules (Figure 2c,d). We detected comparable expression of *GmUOX2* in wild‐type and mutant nodules (Figure 2d). However, we detected no *GmUOX1* expression in mutant nodules, consistent with the chromosome 10 deletion we identified from our CGH and cosegregation analyses (Figure 2a,b). To provide biochemical evidence that GmUOX1 is indeed involved in the catabolism of uric acid during ureide production in soybean nodules, we compared the levels of uric acid in wild‐type and mutant nodules. We found that mutant nodules accumulated very high levels of uric acid, while uric acid was undetectable in wild‐type nodules (Figure 2e).

### 3.2 Fast neutron‐induced mutation of *GmUOX1* is associated with early nodule senescence

**FIGURE 3:**
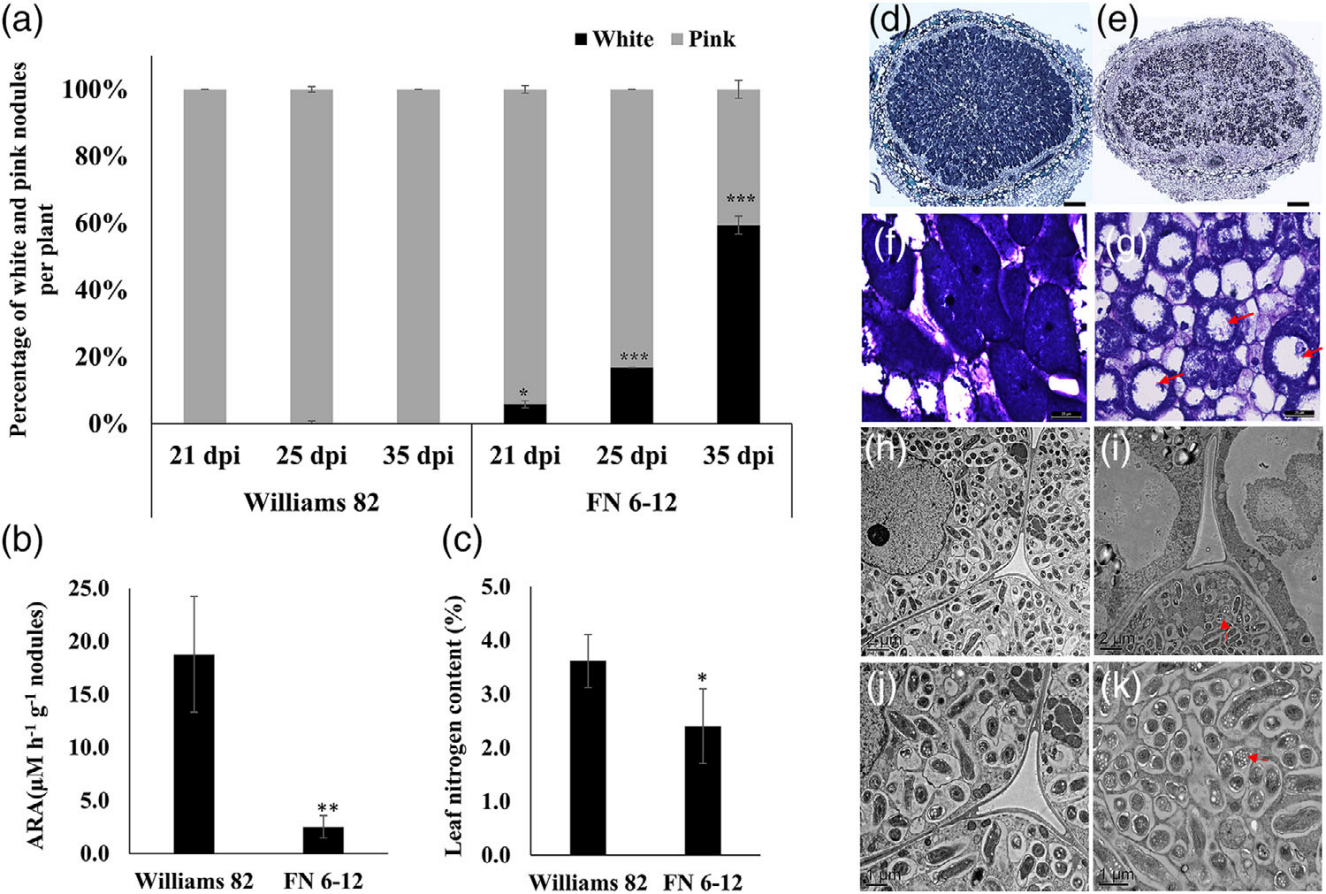
Early nodule senescence and structure of root nodules formed by wild type and FN 6‐12 mutant plants. (a) Percentage of pink and white nodules produced per plant (*n* = 15) by ‘Williams 82’ and FN 6‐12. Data represent mean values ± SD. *(*P* ≤ .05), **(*P* ≤ .01), and *** (*P* ≤ .001) indicate significant differences (Student's *t* test) between the two genotypes. (b) Acetylene reduction assay on 4‐wk‐old nodules from Williams 82 and FN 6‐12 plants. Mean values ± SD for *n* = 4 are shown. ** (*P* ≤ .01) indicates significant differences (Student's *t* test) between two genotypes. (c) Leaf nitrogen content of Williams 82 and FN 6‐12. Mean values ± SD for *n* = 4 are shown. * (*P* ≤ .05) indicates significant differences (Student's *t* test) between two genotypes. (d–g) Representative light microscopy photographs of toluidine blue‐stained nodule sections from (d, f) wild type and (e, g) mutant plants at 25 d after inoculation. Red arrows in (g) indicate symbiosome degradation. (h–k) Representative transmission electron microscopy photographs of (h, j) wild‐type nodule packed with intact symbiosomes and (i, k) mutant nodule with degraded symbiosomes. Red arrows in (h) and (j) indicate highly accumulated poly‐β‐hydroxybutyrate (PHB) in bacteroids of mutant nodule. Bars represent (d, e) 400 μm, (f, g) 25 μm, (h, i) 2 μm, and (j, k) 1 μm

Nodules formed on any root are heterogeneous, with older nodules found near the root crown and younger ones found on younger tap and lateral roots. As mentioned above, white nodules on mutant FN 6‐12 roots were found mostly near the root crown (i.e., older nodules), indicative of early nodule senescence. To determine if this is indeed the case, we examined nodules produced on wild‐type and *gmuox1* roots over a time course. We found that both genotypes formed pink (fully developed) or light pink (developing) nodules at 19 dpi (data not shown). However, the formation of white or greenish‐brown nodules in mutant *gmuox1* roots gradually increased at later time points, that is, at 21, 25, and 35 dpi (Figure 3a). At 35 dpi, ∼60% of FN 6‐12 mutant nodules were white or greenish brown. The formation of white nodules was not observed in all the time points examined in the wild‐type roots (Figure 3a). Thus far, we attributed the Fix^−^ phenotype of *gmuox1* nodules to their white coloration because of the lack of leghemoglobin production. To confirm that the *gmuox1* nodules are indeed unable to fix atmospheric N_2_, we performed acetylene reduction assay to assess the nitrogenase activity of the wild‐type and *gmuox1* nodules. Consistent with reduced levels of leghemoglobin in *gmuox1* nodules, nitrogenase activity was decreased sevenfold in these nodules compared with the wildtype at 28 dpi (Figure 3b). Likewise, *gmuox1* leaves from plants grown in low fertility soil exhibited significantly reduced nitrogen content compared with wild‐type leaves (Figure 3c).

To determine if the exogenous addition of nitrogen can reverse early nodule senescence in *gmuox1* roots, mutant plants were grown under supplementation of 5 μM urea in the watering solution. As controls, wild‐type plants were also grown under similar conditions. Urea was previously reported as a nitrogen source that did not strongly inhibit nodule formation or nitrogen fixation as other nitrogen sources, such as nitrate and ammonium, do (Paradiso et al., 2015; Vigue et al., 1977). As predicted, leaf chlorosis in *gmuox1* plants was reversed by exogenous urea and wild‐type nodules remained pink in color. However, although *gmuox1* plants exhibited no nitrogen deficiency chlorosis, early nodule senescence persisted in mutant roots (Supplemental Figure S4).

To further investigate the biological function of *GmUOX1* during nodule development, nodule sections were stained with toluidine blue and observed under light microscopy. Infected cells of wild‐type nodules at 25 dpi were filled with toluidine blue‐stained bacteroids (Figure 3d,f). In contrast, the infected cells of *gmuox1* nodules were less densely stained and contained large vacuoles (Figure 3e,g). Infected cells of *gmuox1* nodules were also significantly smaller in size than wild‐type nodules. To determine the nodulation defects in *gmuox1* at the cellular level, ultrathin nodule sections were observed by TEM. Consistent with the light microscopy results, infected cells of wild‐type nodules were filled with symbiosomes, new compartments within infected cells harboring bacteroids surrounded by the symbiosome membrane. Symbiosomes typically contain one to five bacteroids (Figure 3h,j). Wild‐type‐like symbiosomes were also observed in infected cells of pink *gmuox1* nodules (data not shown). However, infected cells of white *gmuox1* nodules showed deteriorated or aggregated bacteroids with no discernable symbiosome membranes, increased formation of PHB granules and collapsed infected cells with degraded cytoplasm (Figure 3i,k). Therefore, the degradation of bacteria in the infected cell was correlated to the Fix^−^ phenotype in the mutant line.

**FIGURE 4:**
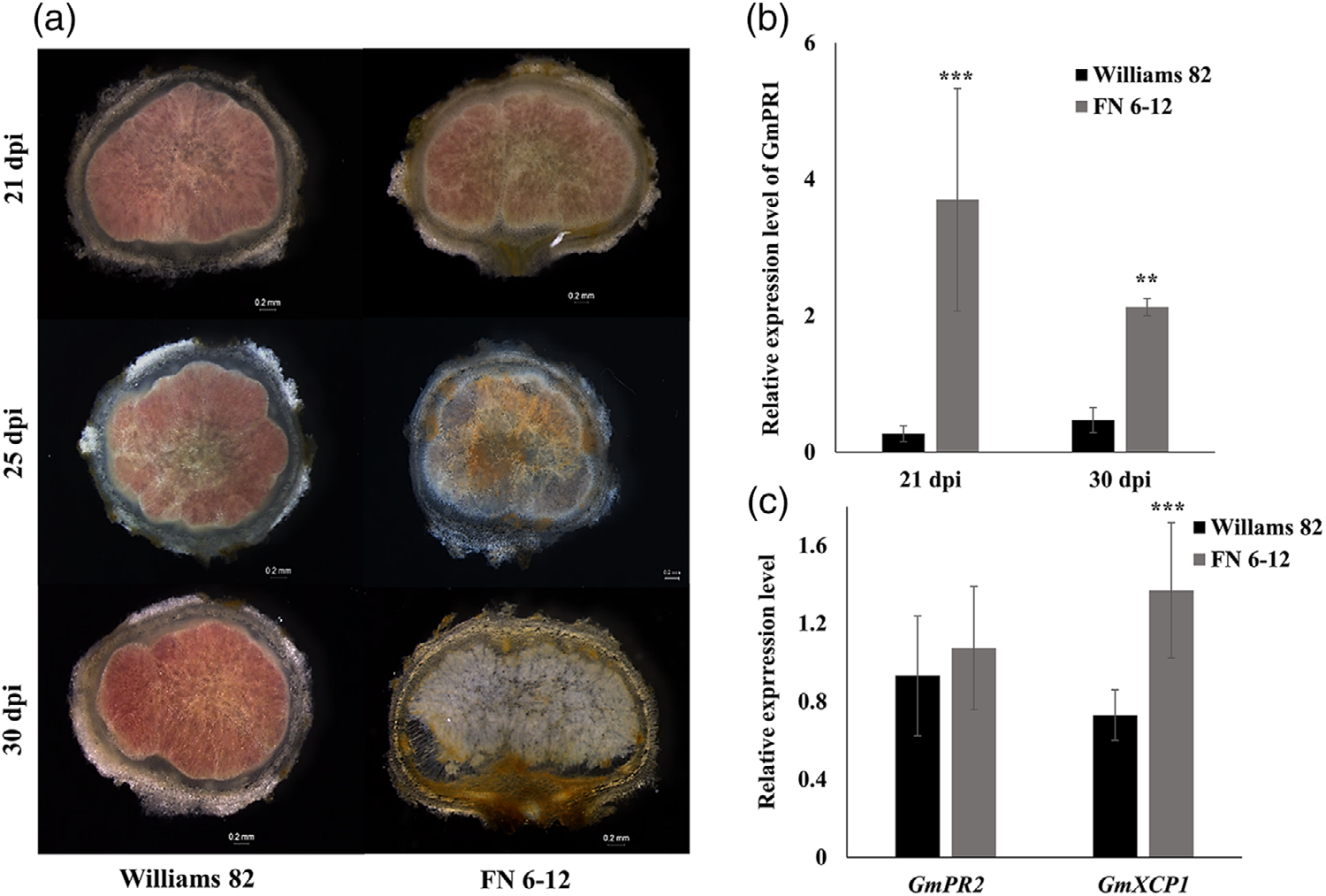
Nodule morphology and expression levels of senescence and defense genes in developing soybean nodules. (a) Vibratome sections of 21, 25, and 30 d after inoculation (dpi) nodules showing early senescence and production of brown pigmentation in the mutant (right panels) but not in wild type (left panels) nodules. (b, c) Expression levels of (b) *Pathogenesis‐Related 1* (*GmPR1*), (c) *Pathogenesis‐Related 2* (*GmPR2*), *and Cysteine protease 1* (*GmXCP1*) in ‘Williams 82’ and FN 6‐12 nodules at 21 dpi. Mean values ± SD for *n* = 4 are shown. ** (*P* < .01), *** (*P* < .001) indicate significant differences (Student's *t* test) between two genotypes

Brown pigmentation in root nodules is often associated with defense reactions and senescence (Berrabah et al., 2014b; Horvath et al., 2015; Pislariu et al., 2019). To determine if brown pigments are produced in *gmuox1* nodules, vibratome sections of wild‐type and *gmuox1* nodules at 21, 25, and 30 dpi were observed. Indeed, brown pigmentation was produced in senescent *gmuox1* nodules but not in wild‐type nodules (Figure 4a). We then analyzed the transcriptional expression of senescence‐ or defense‐related genes at 21 dpi by qRT‐PCR. The expression of soybean *PATHOGENESIS‐RELATED PROTEIN 1* gene *GmPR1* (*Glyma15g06790*) was strongly expressed at the early stage of mutant nodule development, that is, at 21 dpi (13‐fold higher than wild‐type nodule) and remained high at 30 dpi (Figure 4b). However, the expression level of *GmPR2* (*Glyma03g28850*) was not changed in mutant nodules compared with those of the wild‐type nodules (Figure 4c). In addition, the expression level of a *Cysteine protease 1* (*XCP1*, *Glyma17g35720*), a marker for soybean nodule senescence (Alesandrini et al., 2003; van Wyk et al., 2014) was significantly induced in the mutant nodule at 21 dpi compared with the wild‐type nodule (Figure 4c).

Taken together, the observed symbiotic phenotypes of *gmuox1* nodules are consistent with early developmental senescence and the activation of defense‐like responses in host‐plant cells.

### 3.3 Targeted editing of soybean *GmUOX1* by CRISPR/Cas9

**FIGURE 5:**
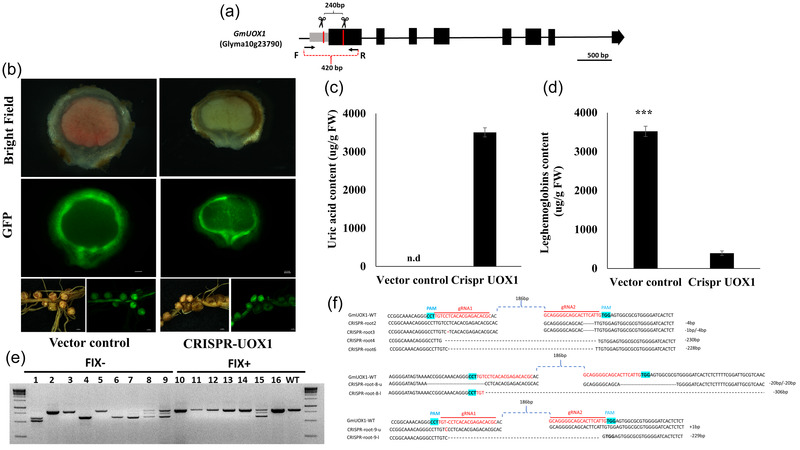
CRISPR/Cas9‐mediated gene editing of *GmUOX1* and symbiotic phenotypes of nodules on transgenic hairy roots. (a) Schematic diagram of dual sgRNAs (scissors) targeting the *GmUOX1* coding sequences. Arrows indicate polymerase chain reaction (PCR) primers used to evaluate induced mutations in transgenic roots. (b) Bright field (top and bottom left panels) and green fluorescent protein (GFP) (middle and bottom right panels) images of nodules formed on transgenic soybean hairy roots showing pink wild‐type nodules vector control and white nodules on homozygous *gmuox1* roots (CRISPR‐UOX1). (c) Uric acid and (d) leghemoglobin levels in vector control and *gmuox1* nodules at 30 d after inoculation (dpi). Mean values ± SD for *n* = 6 are shown in (c) and (d). ***(*P* < .001) indicate significant differences (*t* test) between two genotypes. FW, fresh weight; n.d , not detectable. (e) PCR genotyping of transgenic roots showing Fix^−^ (lanes 1–9) and Fix^+^ (lanes 10–16) nodule phenotypes. Lane WT, ‘Williams 82’. (f) Sequencing of a subset of the PCR amplicons shown in (e) that were derived from Fix^−^ transgenic hairy roots. The root number corresponds to lane number in (e). In cases where two prominent PCR bands were detected, ‘u’ and ‘l’ indicate sequencing of upper and lower band, respectively

The chromosome 10 deletion in the mutant line FN 6‐12 encodes 36 genes including *GmUOX1*. To confirm that the Fix*^−^* phenotype is indeed a result of *gmuox1* deletion and not to codeleted genes in chromosome 10, we used CRISPR/Cas9 mutagenesis to mutate *GmUOX1* by *Agrobacterium rhizogenes*‐mediated hairy root transformation. The dual guide RNAs (gRNAs) system with GFP as selection marker for transformed roots was used (Supplemental Figure S5). The specific target sites were designed at the 5′ untranslated region and the first exon of *GmUOX1* gene to reduce the off‐target effect on *GmUOX2* (Figure 5a). The two target protospacer adjacent motif sites, spaced 240 bp apart, were designed to create defined deletions, which can be detected by mobility shift of PCR amplicons by gel electrophoresis. We examined nodules on transgenic roots transformed with *GmUOX1* gRNAs at 30 dpi and observed that 54 of the 100 roots analyzed (54%) formed white nodules. In contrast, nodules on vector‐only control roots were all Fix^+^, that is, all pink in color (Figure 5b). Compared with control nodules, the CRISPR nodules were smaller and accumulated significantly higher levels of uric acid (Figure 5b,c) and lower levels of leghemoglobin (Figure 5d), which is consistent with predicted phenotypes associated with *gmuox1* null mutation.

To determine if the production of Fix^−^ nodules is associated with CRISPR‐edited *GmUOX1*, we extracted genomic DNA from transgenic roots harboring Fix^+^ or Fix^−^ nodules and amplified a *GmUOX1* DNA segment containing Targets 1 and 2 by PCR using primers flanking the target sites (F and R in Figure 5a). Our results showed that some Fix^−^ roots (Samples 1, 4, 6, and 7) showed only low mobility bands, consistent with DNA deletions because of two double‐strand breaks at Targets 1 and 2 (Figure 5e). However, we also observed PCR amplicons from Fix^−^ roots with similar mobility as wild‐type roots (Samples 2, 3, and 5), as well as mixed amplicons for both wild‐type and deleted DNA fragments (Samples 8 and 9) (Figure 5e). Sequencing of PCR amplicons showed that the low mobility bands contained deletions spanning Targets 1 and 2 (i.e., Samples 4 and 6 in Figure 5e,f), whereas wild‐type‐appearing bands showed small deletions in close proximity to either or both target sites (i.e., Samples 2 and 3 in Figure 5e,f). Likewise, sequencing of PCR amplicons from Fix^−^ roots showing both wild‐type and deleted bands showed biallelic mutations in *GmUOX1* (Samples 8 and 9 in Figure 5e,f). In contrast, most PCR amplicons from Fix^+^ roots showed wild‐type bands (Figure 5e), which, when sequenced, all had no detectable mutation (data not shown). In cases where we observed both wild‐type and deleted bands on Fix^+^ roots (e.g., Sample 15, Figure 5b), sequencing of amplicons showed these roots to be heterozygous or chimeric for mutations in *GmUOX1*. To determine if *GmUOX2* was targeted because of its high sequence identity to *GmUOX1*, *GmUOX2* sequences were PCR amplified in Fix^−^ nodules using *GmUOX2*‐specific primers. We observed no gel‐shifted *GmUOX2* PCR bands (Supplemental Figure S6a) and no mutation was observed in *GmUOX2* after sequencing of amplicons (data not shown). In addition, the expression level of *GmUOX2* did not change in these transgenic roots (Supplemental Figure S6b).

In summary, these results show that we obtained multiple mutant alleles in *GmUOX1* using CRISPR/Cas9 targeted gene editing. Moreover, homozygous or biallelic CRISPR mutants phenocopied the nodule phenotypes observed in the FN *gmuox1* mutant, that is, reduced leghemoglobin accumulation, reduced nodule size, increased levels of uric acid, and production of white nodules. Therefore, the early nodule senescence phenotype described above for the mutant FN 6‐12 indeed is due to the deletion of *GmUOX1*.

### 3.4 Defective nodule development in CRISPR/Cas9‐edited *GmXDH1* roots

**FIGURE 6:**
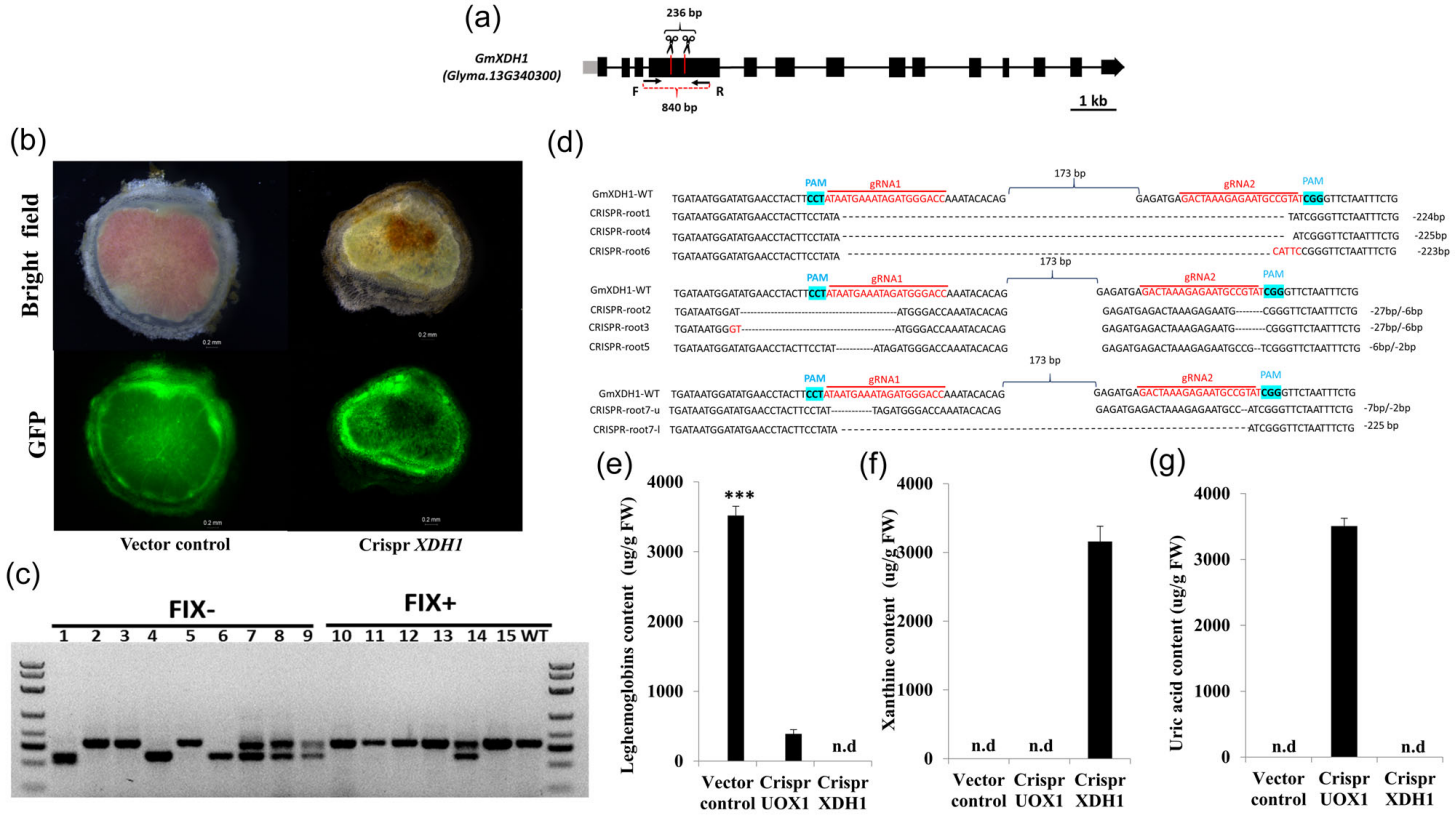
CRISPR/Cas9‐mediated gene editing of *GmXDH1* and symbiotic phenotypes of nodules on transgenic hairy roots. (a) Schematic diagram of dual sgRNAs (scissors) targeting the *GmXDH1* coding sequences. Arrows indicate polymerase chain reaction (PCR) primers used to evaluate induced mutations in transgenic roots. (b) Bright field (top panels) and green fluorescent protein (GFP) (bottom panels) images of nodules formed on transgenic soybean hairy roots showing pink wild‐type nodules (vector control, left panels) and *gmxdh1* (right panels) nodules. (c) PCR genotyping of transgenic roots showing Fix^−^ (lanes 1–9) and Fix^+^ (lanes 10–15) nodule phenotypes. Lane WT, ‘Williams 82’. (d) Sequencing of a subset of the PCR amplicons shown in (c) that were derived from Fix^−^ transgenic hairy roots. The root number corresponds to lane number in (c). In cases where two prominent PCR bands were detected, ‘u’ and ‘l’ indicate sequencing of upper and lower band, respectively. (e) Leghemoglobin, (f) xanthine, and (g) uric acid content in wild‐type (vector control), *gmuox1* (Crispr UOX1), and *gmxdh1* (Crispr XDH1) nodules at 30 d after inoculation (dpi). Mean values ± SD for *n* = 4 are shown in (e–g). *** (*P* < .001) indicates significant differences between genotypes (ANOVA, Tukey's HSD test). FW, fresh weight; n.d, not detectable

**FIGURE 7:**
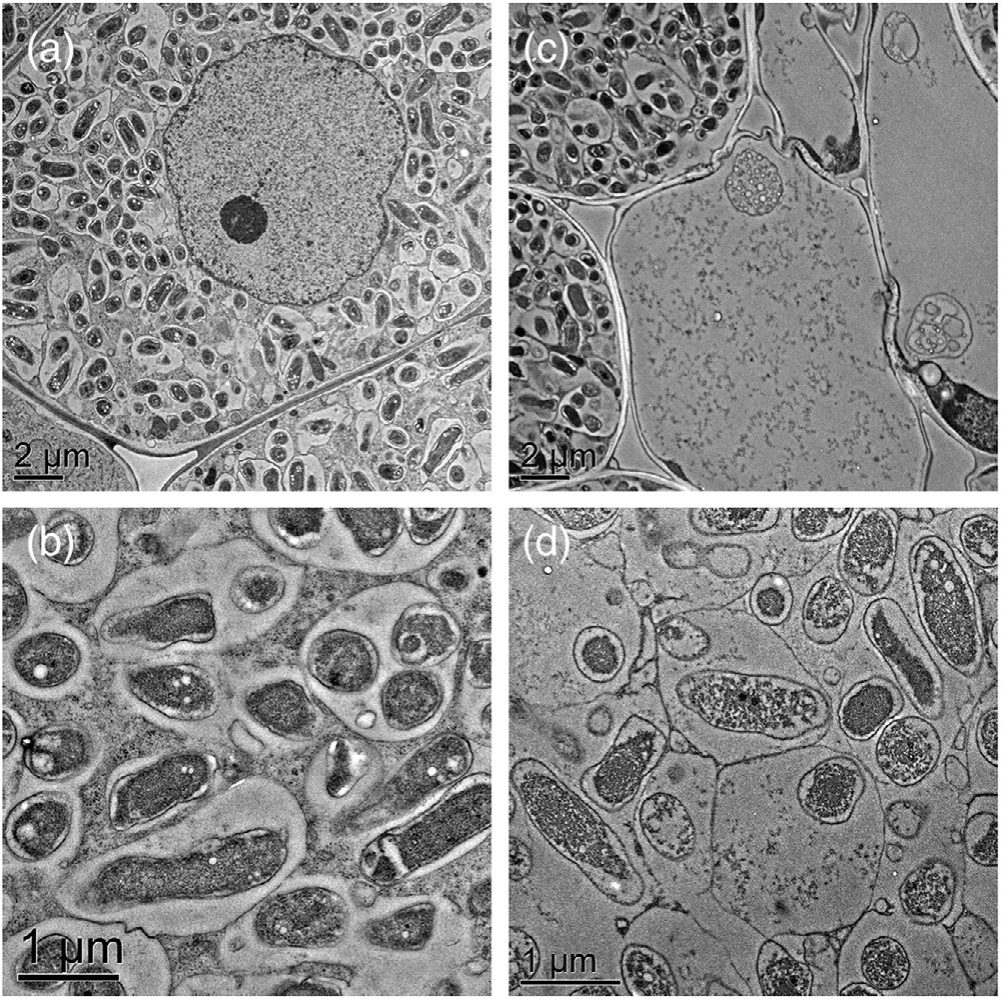
Ultrastructure of root nodules of wild‐type and CRISPR‐edited *gmxdh1* plants at 28 d after inoculation. (a, b) Representative transmission electron microscopy (TEM) images of healthy, nitrogen‐fixing, wild‐type nodules packed with bacteroids. (c, d) Representative TEM images of *gmxdh1* nodules showing degradation of bacteriods. Bars represent 2 μm (a, c) and 1 μm (b, d)

To determine if defective nodule development is due to a nonfunctional *GmUOX1* per se or to defective ureide biosynthesis in general, we identified the soybean genes XDH for targeted editing by CRISPR/Cas9 in hairy roots. Xanthine dehydrogenase is upstream of UOX in the ureide biosynthetic pathway and catalyzes the conversion of xanthine to uric acid (Supplemental Figure S1). There are two paralogous *GmXDH* genes in the soybean genome, *Glyma13g41520* (*GmXDH1*) and *Glyma15g03870* (*GmXDH2*), with *GmXDH1* the only gene copy that is highly expressed in nodules (Supplemental Figure S3). We therefore designed two sgRNAs targeting the first exon of *GmXDH1* (Figure 6a) to create deletions that can facilitate the identification of CRISPR‐edited hairy roots. We used the same CRISPR/Cas9 vector system as mentioned above for *GmUOX1* and used GFP fluorescence to identify transformed roots. Examination of nodules at 30 dpi showed that out of 124 transgenic roots that expressed GFP, 86 (∼69%) formed small white and brown nodules. In contrast, nodules on vector‐only control roots were all Fix^+^ (Figure 6b). Transgenic roots were genotyped for *GmXDH1* as described above for *GmUOX1*, that is, by PCR and sequencing of PCR amplicons. Our genotyping data showed that all Fix^−^ roots harbored homozygous (Samples 1–6 in Figure 6c,d) or biallelic (Samples 7, 8, and 9 in Figure 6b,c,d) mutations in *GmXDH1*. In contrast, Fix^+^ nodules were homozygous wild‐type or heterozygous *gmxdh1* mutants (Sample 14; Figure 6b,c,d) for *GmXDH1*. Like the *gmuox1* nodules, *gmxdh1* nodules were smaller and contained significantly reduced leghemoglobin compared with those formed on vector‐only control roots (Figure 6b,e). *gmxdh1* nodules accumulated high amounts of xanthine, and no trace of the uric acid was detected in the *gmxdh1* nodules (Figure 6f,g). They were consistent with the predicted function of GmXDH1 in converting xanthine to uric acid during ureide biosynthesis in soybean nodules (Figure 6f,g; Supplemental Figure S1). Further examination of nodule morphology by TEM showed that infected cells of the wild‐type nodules were filled with intact symbiosomes (Figure 7a,b) , while infected cells of *gmxdh1* mutant nodules were degraded (Figure 7c,d) similar to those observed in *gmuox1* nodules (Figure 3e,g,i,k). Lastly, like *gmuox1* nodules, *gmxdh1* nodules exhibited brown coloration indicative of increased defense response. Taken together, our results showed that CRISPR‐induced knockout mutations in *GmXDH1* resulted in Fix*^−^* nodules. Together, these data indicate that, like *GmUOX1*, functional *GmXDH1* is required for nodule persistence in soybean roots.

## 4 DISCUSSION

*GmUOX1* encodes URICASE, the key enzyme that converts uric acid to ureides, the major form of fixed nitrogen that is transported from nodules to other parts of the plant. Knockout *gmuox1* soybean plants were able to form nodules, but the nodules senesced prematurely. At 21 dpi, 6% of total nodules formed on *gmuox1* roots showed a Fix^−^ phenotype, which rapidly increased up to ∼60% of total crown nodules at 35 dpi (Figures 1b and 3a; Supplemental Figure S2). In contrast, nodules developed on wild‐type roots did not show signs of senescence at 35 dpi (Figures 1b and 3a; Supplemental Figure S2). In addition, the expression level of a *Cysteine protease 1* (*XCP1‐ Glyma17g35720*), a marker for soybean nodule senescence (Alesandrini et al., 2003; van Wyk et al., 2014) was significantly induced in the mutant nodules at 21 dpi (Figure 4c). Microscopic observations of mutant nodules showed that bacteroids contained a higher number of PHB granules (Figures 3i,k) and symbiosomes were degraded starting at ∼21 dpi (Figures 3a,g,i). The content of PHB in bacteroids is dependent on the relative rates of synthesis and degradation during active nitrogen fixation (Lodwig & Poole, 2003). High accumulation of PHB in bacteroids is a feature of senescing nodules in different legumes (Hernández‐López et al., 2018; Li et al., 2015; Strodtman et al., 2018), which could be due to a variety of causes, including a shift in the redox state of the cell, shifts in the nitrogen–carbon balance, or disruption in the ability to degrade and utilize PHB. Lastly, the higher number of nodules produced in *gmuox1* roots (Figure 1c) is consistent with previous reports on Fix^−^ mutants in legumes (Bourcy et al., 2013; Wang et al., 2016; Xi et al., 2013), which was suggested to compensate for the fixation deficiency in the mutant roots. Overall, these data provided genetic and biochemical evidence on the importance of *GmUOX1* in the successful establishment of functional nodules in soybean.

Although URICASE is a critical enzyme in the formation of ureides in soybean and other legumes, it also functions in purine catabolism in other organisms. In Arabidopsis, high accumulation of uric acid in the developing embryo of *atuox* plants reduced the germination rate and inhibited cotyledon development (Hauck et al., 2014). The high accumulation of uric acid in *atuox* mutants apparently blocked peroxisome maintenance, impairing seedling establishment. Likewise, extensive accumulation of uric acid in serum could lead to several diseases, including gout, coronary heart disease, hypertension, and neurodegenerative disorders in human, or high lethality in mutant mice lacking *UOX* (Rock et al., 2013; Wu et al., 1994). Reduced production of uric acid by inhibiting XDH activity increased the germination rate in Arabidopsis and rescued lethality in mice (Hauck et al., 2014; Wu et al., 1994). Therefore, we hypothesized that the early nodule senescence associated with *gmuox* mutations may be due to the toxic buildup of uric acid in mutant nodules (Figures 2e and 5c). To test this hypothesis, we generated *gmxdh1* hairy roots using CRISPR/Cas9 and characterized the nodules formed. Our results showed that, although uric acid was reduced to nondetectable levels, *gmxdh1* roots produced nodules that senesced early and phenocopied the aberrant nodule phenotypes of *gmuox1* roots (Figures 6b,e,f,g and 7c,d). These results, therefore, suggest that the high accumulation of uric acid in *gmuox1* nodules was not the direct cause of the observed early nodule senescence phenotype. In human, deficiency of XDH leads to accumulate a pure xanthine stone and to acute renal failure (Arikyants et al., 2007). Thus, it is possible that the high levels of xanthine content in nodules was toxic and caused the early senescence of *gmxdh1* nodules. However, unlike uric acid toxicity, to our knowledge, xanthine toxicity has not been reported in plants so far. In Arabidopsis, *AtXDH* RNAi or *atxdh1* null mutants exhibited premature leaf senescence (Brychkova et al., 2008; Nakagawa et al., 2007; Soltabayeva et al., 2018). However, this phenotype was reversed by exogenous supplementation of uric acid, allantoin, allantoate, or nitrate, thus indicating that the early leaf senescence phenotype is due to ureide and nitrogen deficiency rather than to overaccumulation of xanthine per se. Therefore, it is likely that the early nodule senescence in *gmuox1* and *gmxdh1* was due to defective purine catabolism and the consequent lack of ureide remobilization in the mutant nodules. Although the cause of the subsequent induction of early nodule senescence in the Fix^−^ nodules is unknown, it is possible that it is due to direct sanctioning of these nodules by the plant as a way of limiting the carbon flowing to nonproductive organs (Kiers & Denison, 2008; Westhoek et al., 2021). At a minimum, the data point to a negative feedback mechanism that exists in nodules resulting in early senescence when nitrogen fixation is disrupted.

Premature or early nodule senescence is not unique to *gmuox1* and *gmxdh1* soybean plants, but rather is a common phenotype of mutant legumes that are able to initiate nodule formation but failed to establish effective N_2_ fixation (Banba et al., 2001; Berrabah et al., 2014b; Bourcy et al., 2013; Kawaguchi et al., 2002; Kumagai et al., 2007). For example, early senescence was observed in the well‐characterized *sen1 Lotus japonicus* mutant that completely lacked N_2_ fixation due to defective rhizobial differentiation to bacteroids (Hakoyama et al., 2011; Kawaguchi et al., 2002; Suganuma et al., 2003). Previous studies have also shown that early nodule senescence is not simply due to nitrogen deficiency caused by ineffective nitrogen fixation per se (Banba et al., 2001), but rather a reflection of active induction of plant defense response to non‐fixing endosymbionts that confer no benefit to the host plants (Berrabah et al., 2014a; Bourcy et al., 2013; Horváth et al., 2015; Krishnan et al., 2016; Wang et al., 2016). The data presented here support this notion. First, addition of exogenous 5 μM urea reversed leaf chlorosis, but not early nodule senescence, in *gmuox1* plants (Figure S4). The added urea had no negative effect on nodule development in wild‐type roots, consistent with previous reports that this nitrogen source did not strongly inhibit nodule formation nor nitrogen fixation as other nitrogen sources, *e.g*., nitrate and ammonium (Paradiso et al., 2015; Vigue et al., 1977). Secondly, consistent with the activation of plant defense response, *gmuox1* and *gmxdh1* nodules showed dark brown pigmentations that were not observed in wild type nodules (Figures. 1b, 4a, 5, 6, Figure S2, 4). Production of brown pigmentation is an indicator of the presence of polyphenolic compounds, which are often associated with a defense response (Berrabah et al., 2014a; Horváth et al., 2015; Pislariu et al., 2019). In addition, the pathogen response *GmPR1* gene, but not *GmPR2*, was significantly induced in the *gmuox1* nodules at 21 and 30 dpi (Figure 4b,c), again indicating that defense response was activated in the mutant nodules.

The soybean genome is allotetraploid with nearly 75% of the genes present in multiple copies (Schmutz et al., 2010). However, ∼50% of gene paralogs in soybean were found to be differentially expressed and thus had undergone expression subfunctionalization (Roulin et al., 2013). The two paralogous GmUOX and GmXDH proteins share 99 and 98% amino acid sequence identity, respectively. *GmUOX1* is expressed strongly in nodules but not in other tissues (Figure 2c; Supplemental Figure S3). In contrast, *GmUOX2* is weakly expressed in nodules but is expressed at higher levels in other tissues compared with *GmUOX1* (Figure 2d; Supplemental Figure S3). A similar tissue‐specific expression subfunctionalization of *GmXDH* genes exist, with GmXDH1 the major functional isoform in root nodules. Although the presence of the ureide biosynthesis pathway is well documented in legume plants, identification of the enzymes involved is largely based on predicted genome annotations with little direct genetic or biochemical evidence to confirm such predictions. In this study, we showed the critical function of de novo purine catabolism in successful nodule development and identified GmUOX1 and GmXDH1 as the major isoforms involved in the biosynthesis of ureides in soybean nodules. Moreover, we used FN and CRISPR/Cas9 mutagenesis to obtain stable and transient mutant alleles in soybean hairy roots, respectively. We previously reported on the development of dual gRNA CRISPR/Cas9 reagents for high‐frequency mutagenesis in soybean through *Agrobacterium*‐mediated stable transformation (Do et al., 2019; Nguyen et al., 2021). Here, we used dual gRNA CRISPR/Cas9 to obtain mutant alleles of *GmUOX1* and *GmXDH1* in soybean hairy roots. One initial concern we had with this approach is the likelihood of low‐frequency homozygous mutations, a necessary prerequisite for observing traits associated with recessive mutant alleles. However, we were able obtain 54–64% of transgenic roots that produced white nodules, indicating that indeed we can obtain homozygous mutant roots at relatively high frequency. It is very likely that higher frequency of mutations can be obtained if additional gene‐specific gRNAs are employed, that is, more than the two gRNAs we used in this study. Generating stable transformants in plants is costly and time consuming, especially in crops that are recalcitrant to transformation such as soybean. The data presented here demonstrate the utility of the CRISPR/Cas9 system for studying root‐associated genetic traits when coupled with hairy root transformation.

## AUTHOR CONTRIBUTIONS

Cuong X. Nguyen: Conceptualization, Data curation, Formal analysis, Investigation, Methodology, Resources, Software, Validation, Visualization, Writing‐original draft. Alice Dohnalkova: Methodology. C. Nathan Hancock: Methodology. Kendall R. Kirk: Methodology. Gary Stacey: Funding acquisition, Writing‐original draft. Minviluz G. Stacey: Funding acquisition, Project administration, Supervision, Writing‐original draft.

## CONFLICT OF INTEREST

All authors state no conflicts of interest concerning this manuscript.

## Supporting information

Supplemental Figure S1 Nitrogen fixation and assimilation in soybean nodules.

Supplemental Figure S2. Symbiotic phenotypes of the mutant line K14 at 30 days after inoculation with *B*. *japonicum* strain USDA 110

Supplemental Figure S3. Expression profile of *GmUOX1, GmUOX2, GmXDH1 and GmXDH2* in different Williams 82 tissues.

Supplemental Figure S4. Symbiotic phenotypes of Williams 82 (a, c) and FN 6‐12 (b, d) plants grown with exogenous addition of 5 μM urea.

Supplemental Figure S5. Schematic diagram of the dual gRNAs CRISPR/Cas9 system used for hairy root transformation in soybean.

Supplemental Figure S6. Off‐target detection by PCR genotyping (a) and qPCR (b) for *GmUOX2* from transgenic roots showed Fix*^‐^* nodule phenotypes

Supplemental Table S1. Primers used in this study.
Table S2. Segregation analysis of F_2_ progenies derived from Williams 82 X FN 6‐12 genetic crosses.
